# Controls on explosive-effusive volcanic eruption styles

**DOI:** 10.1038/s41467-018-05293-3

**Published:** 2018-07-19

**Authors:** Mike Cassidy, Michael Manga, Kathy Cashman, Olivier Bachmann

**Affiliations:** 1 Department of Earth Sciences, University of Oxford, Oxford, South Parks Road, OX1 3AN UK; 2 Department of Earth and Planetary Science, University of California, Berkeley, 94720-4767 CA USA; 3 School of Earth Sciences, University of Bristol, Wills Memorial Building, Queens Road, Bristol, BS8 1RJ UK; 4 Institute of Geochemistry and Petrology, NW E 83.3 Clausiusstrasse, 25 8092 Zürich, Switzerland

## Abstract

One of the biggest challenges in volcanic hazard assessment is to understand how and why eruptive style changes within the same eruptive period or even from one eruption to the next at a given volcano. This review evaluates the competing processes that lead to explosive and effusive eruptions of silicic magmas. Eruptive style depends on a set of feedback involving interrelated magmatic properties and processes. Foremost of these are magma viscosity, gas loss and external properties such as conduit geometry. Ultimately, these parameters control the speed at which magmas ascend, decompress and outgas en route to the surface, and thus determine eruptive style and evolution.

## Abstract (web summary)

Eruptive styles at a single volcano may transition from explosive to effusive behaviour (or vice versa) at any given time. This review examines the underlying controls on eruptive styles such as magma viscosity, degassing and conduit geometry at volcanoes with silicic compositions.

## Introduction

With the increasing global population and stress on natural resources, volcanoes threaten more lives every day. Explosive volcanic eruptions can have devastating societal impacts on nearby populations, covering entire countries in ash, ruining crops, killing livestock and causing a huge loss of human life. These eruptions can also have global effects, with the potential to impact air traffic, air quality, global temperatures and biogeochemical cycles. Conversely, lava flow or dome-forming (effusive) eruptions are generally less hazardous, with impacts focused in the area immediately surrounding the volcano, although eruptions of large mafic lava flows can destroy property and may have adverse effects on regional air quality^1^. Therefore, the style of volcanism dictates the types of hazards posed by a volcano. An important problem is that any one volcano can erupt either explosively or effusively, and a single eruptive episode may include multiple and rapid changes in eruptive style^2^. Out of the 106 eruptions greater than or equal to Volcano Explosivity Index (VEI) 3 since 2000, 61% of these comprised both effusive and explosive activity (Global Volcanism Program, 2013^3^). Additionally, our current understanding of the geophysical, geodetic and geochemical signals detected by volcano monitoring does not provide either an adequate framework to reliably forecast the initial eruption style and size, or the temporal evolution of eruptive activity. This ambiguity limits the ability of authorities to prepare for and mitigate against volcanic hazards. It is thus critical to understand the factors that control whether a volcano erupts effusively or explosively, and to integrate this information into models that provide realistic eruption scenarios. This goal is considered to be one of the three grand challenges in volcano science^4^.

**Fig. 1:**
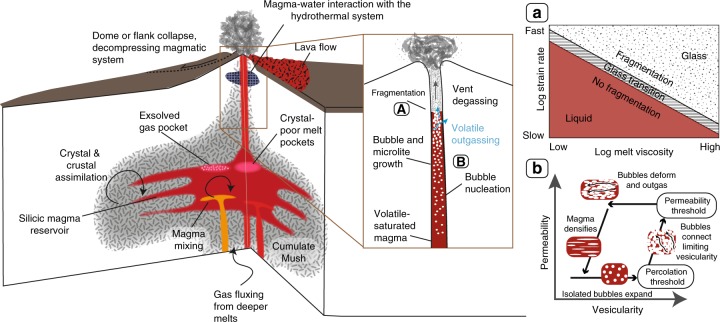
Annotated volcano schematic, illustrating the range of processes that can affect eruptive style from storage to surface, prior to or during volcanic eruptions. **a** Shows how fragmentation is a function of the melt viscosity and strain rate. Fast strain rates (i.e., high decompression rates) favour brittle fragmentation as it passes the glass transition for a given viscosity. At slow strain rates, magma generally behaves as a liquid. Figure adapted from Gonnermann and Manga^105^. **b** Shows the cycle of volatile outgassing, from nucleation, coalescence to densification from permeability and porosity data in Rust and Cashman^80^, figure reproduced from Cashman and Sparks^5^, under fair usage terms

In this review, we focus on the shall-owest parts of the magmatic system, from the subvolcanic magma storage to conduit flow and surface events (upper ~10 km; Fig. 1 and Table 1). In this low pressure environment, processes such as crystal and bubble growth (Fig. 1b) affect the permeability, rheology, extent of outgassing, and fragmentation depth and mechanisms (Fig. 1b and Table 1) of the magma in the plumbing system. Furthermore, even processes that occur at the surface such as dome collapse, earthquakes, ice melting or landslides can exert pressure changes that may propagate downwards altering the stress fields and affecting the decompression rate (Fig. 1 and Table 1). Albeit not discussed in detail, processes occurring deeper in the crust (below ~10 km) can also exert important controls on properties and parameters such as magma production rate, density, buoyancy, composition, volatile contents and viscosity. We stress that these properties can vary during storage and ascent (for instance, through the process of magma crystallization and differentiation, when the silica content of the magma, viscosity of the melt and residual volatile contents increase) and may play non-trivial roles in controlling eruptive styles. Indeed, processes with roots in the mid-to-upper crust, such as magma recharge (inducing mixing/mingling) and crustal assimilation, may significantly alter many of the critical magma (intrinsic) properties or parameters, including temperature, rheology, volatile solubility, magmatic overpressure, dissolved and exsolved gas content, bubble and crystal content, and magma ascent rate (Table 1). This contribution focuses on intermediate to more silicic compositions, i.e., high viscosity magmas. A caveat to this approach is that whole-rock compositional classifications can be misleading in the frame of eruptive style, since crystal-rich magmas, even of mafic compositions (basalts and andesites), may have more silicic ‘melt’ compositions, and taken with their crystal-rich assemblage, can make these magmas more rheologically analogous to silicic magmas^5,6^.

**Table 1:** Here, various magma properties (intrinsic) or extrinsic parameters (highlighted in bold) are given, which have been suggested to affect volcano explosivity. Their controls are given along with brief explanations of how this can affect eruptive style. Not all these references will be discussed in the text, but this serves as a reference table for the reader to seek out more detail. Deeper controls on eruptive style, not addressed in this table, include magma composition, buoyancy, and magma production rate

Properties/parameters	What controls this?	How does this affect eruptive style?
**Tectonic regime/stress field**	Regional faulting, edifice load, pressure differential between magma and surface	Alterations to the stress field such as unloading via flank/dome collapse can alter magma ascent rates^40,225^, e.g., rapid decompression from 12 MPa at Mt St Helens leads to explosive fragmentation^54^
Magma ascent rate	Stress field, magma buoyancy, volatiles, conduit geometry, chamber overpressure, viscosity	Faster ascent (e.g., >0.1 m/s per second), thus less time for outgassing, generally promotes explosive eruption^37,65,136,182^ (See supplementary data 1 and Fig. 5)
Dissolved and exsolved volatile content	Magma petrogenesis &composition, pressure/depth solubility, saturation degree, gas and magma influx	When volatiles are coupled to the magma, they increase buoyancy and ascent speed, if not lost during ascent^25,26,28,37,133,143,145,226,227^
Magma rheology/viscosity	Magma and volatile composition, temperature, crystal and bubble content, strain rates	High viscosity inhibits release of volatiles promoting closed-system degassing^8,41,228^. Small changes of viscosity by ~0.5 (log Pa/s), may dictate whether an eruption becomes effusive or explosive^18,21^
Magma chamber overpressure	Magma production rate, dome growth/collapse, magma recharge, exsolved volatiles	High overpressures (typically, 0–20 MPa^137^) may drive faster ascent speeds^15,38,73^, but this parameter is not well constrained
Magma temperature	Magma composition, magmatic injections, shear in conduit, time in storage/ascent, latent heat of exsolution and crystallization	Affects the magma viscosity, crystal content and volatile solubility can promote more open-system degassing and effusive^8,71,229^. These studies showed that changes as small as 80 °C can lead to changes between effusive and explosive eruption
**Rate of decompression**	When coupled, the magma ascent rate, speed of release of pressure, unloading events, stress field	Same effects as magma ascent rate, these are normally coupled. However, fast decompression via unloading can lead to rapid downward-propagating fragmentation wave^52,54^. Typical decompression rates of explosive eruptions are: 1 MPa/s and typical effusive rates (0.0001 MPa/s) (Supplemental data 1 and Fig. 5)
**Crustal properties (e.g., conduit wall permeability)**	Regional geology, stress field, fracture networks, anisotropy, discontinuities.	More permeable crust (and isotropic) leads to open-system degassing and thus generally less explosive^36,73,117^. However, the rate of outgassing through conduit walls is several orders of magnitude lower than vertical outgassing loss through conduit shear zones^230^
Porosity and permeability of magma	Brittle deformation, fractures, bubble connectivity, bubble number density, decompression rate, viscosity, crystal content.	Higher porosity and permeability promote more of outgassing and alters fragmentation^56,75,83,88,181,231^. Magma must reach percolation threshold for efficient gas permeability, which ranges from 30 to 78% in vesiculating systems^87^. Magmas may repeatedly fracture and heal thus varying permeability and outgassing^58,77,97,112,232^
Crystal and bubble content	Magma composition and rheology, magma ascent rate, P-T conditions on ascent, hetero vs homogenous bubble nucleation, crystal fractionation and assimilation, magma and gas influx	Affects the viscosity, permeability and efficiency of outgassing^83,88,133–135,137,233^. Buoyancy-driven outgassing is efficient (40–50% volatiles outgas) where crystallinities are between 40–70%^135^. Homogeneous nucleation leads to delayed disequilibrium degassing^48,50^. High bubble number densities leads to less outgassing and more explosive eruptions, e.g., ~10^15^ m^−3^ for Mt St Helens explosive eruptions^234^, and ~5 orders of magnitude less for effusive Soufriere Hills lava dome eruptions^83,197^
**Conduit and vent geometry**	Erosion during explosions, magma accretion on walls, magma rheology, crustal structure and properties	Can affect the ascent speed and the magma extrusion rate at the surface^122,123,235^. Modelling shows that doubling conduit radius from 10 to 20 m increases ascent rate 4 times^45^
**External assimilation (e.g., surface water, carbonates)**	Regional geology, hydrothermal system, geographic setting (e.g., glacial regions)	Explosive expansion and fragmentation of silicate glass^68^. Experiments on rhyolites show higher kinetic energy release by wet compared to dry experiments (>100 m/s different in ejection velocity)^236^. Additional volatiles may come from carbonates, experiments show, carbonate can completely breakdown in <10 min within melts at temperatures of 1200 C and 0.5 GPa^66^
Magma depth/pressure	Density and buoyancy contrast between crust and magma, crustal structure/discontinuities	Affects solubility of different volatiles, buoyancy and affects local stress field, thus altering ascent^138^. Explosive phonolite eruptions derived from magma source from 1–6 km depth and water saturated^35^

An important distinction to make when discussing eruptive style transitions is the time scale over which these occur. Transitions between effusive and explosive volcanism can occur during a single eruptive phase, e.g., during dome growth and collapse episodes, and Vulcanian explosions. Transitions in eruptive style at the same volcano can also occur over several eruptions, e.g., a lava flow and a Plinian eruption separated by a repose interval. Here we highlight that the first type of transition (within single eruptive phase) is dominantly affected by shallow processes (within the conduit <3 km), whereas the second type may also be controlled by conditions and processes within the magma reservoir and early ascent in the conduit (>3–10 km).

The explosive versus effusive issue in volcano forecasting has been approached by many different fields and disciplines, including petrology, geochemistry, fluid dynamics, numerical modelling, gas geochemistry and rock deformation. Recent and often multidisciplinary breakthroughs in this research, however, attribute the controls on eruptive styles to different volcano properties (Table 1). Therefore, we aim to reconcile this complex body of research and to frame discussions of eruptive style for a wide audience. The review describes different causes and feedbacks involved in both explosive and effusive silicic eruptions. We provide data synthesis of magma ascent and decompression rates, we collate the parameters and properties that play a role in eruptive style and include statistics regarding the abundance and timing of effusive–explosive transitions. This review aims to not only to summarize the recent literature, but also to provide ideas for potential new research directions, including ways that the community can link to this body of research to volcano monitoring with the goal of improving the forecasting of explosive and effusive behaviour.

## Factors promoting explosive volcanism

The various properties or parameters that influence volcano explosivity may affect eruptive style in contradictory ways (Table 1). For instance, the injection of magma into the storage reservoir (Fig. 1) may lead to either explosive or effusive eruptions^7,8^. These contradictions exist because there is no single way to generate an explosive eruption. This section describes the processes that may lead to explosive activity, which is reviewed in this section from storage to surface.

### Effusive–explosive transitions between eruptive episodes

Where transitions in eruptive style at the same volcano occur over several eruptions, these changes may be closely linked to the mechanism that triggered the eruption. A common example of eruptive activity driven by processes at depth involves injection of new magma into a subvolcanic reservoir^9–12^. Explosive eruptions can be triggered under these conditions by either heating, which causes convection and vesiculation^9^ or mobilizes crystal-rich magmas^13,14^; addition of volume, which increases the overpressure of the magma system on the confining walls^15^; and/or fluxing of volatiles that increases buoyancy^7,12,16^. However, magma injection may also lead to effusive volcanism, by decreasing the viscosity of the system through heating and resorption of crystals which lower viscosity^8,17^ and reducing the water content, if the influxing magma is water-poor^18^. Reduced viscosity can change the nature of fragmentation (Fig. 1a), inhibiting brittle failure and enhancing non-explosive loss of volatiles. The process of magma mixing is therefore complex and its effect on volcano explosivity is likely dependent on the degree of mixing, presence of exsolved volatiles, extent of volatile and heat transfer and time between mixing and eruption^15^. In these examples, where the same volcano exhibits different styles of eruptions, the explosive eruptions occur when there is limited^8^ or no magma injections^18,19^. Instead, cooling and crystallization of dominantly anhydrous crystals, leading to second boiling^20^ can promote overpressurization in the reservoir, driving faster magma ascent and more explosive activity. Even subtle changes in viscosity alone (~0.5 log Pa/s) in these instances as a result of heating, crystallization or compositional changes may determine the eruptive style^21^.

### Effusive–explosive transitions within single eruptive episodes

**Fig. 2:**
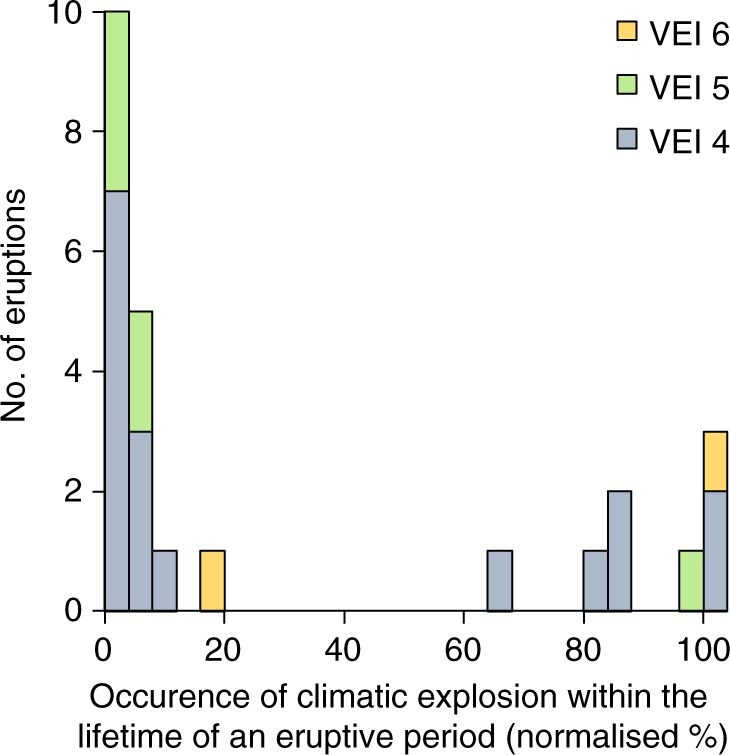
Graph of known eruptive time periods with both climactic explosions (VEI ≥ 4) and dome growth (including crypto-domes) from the Domehaz database^22^. All eruptive time periods (which includes smaller explosions, dome extrusion) have been normalized. Two-thirds of the climactic eruptions occur at the start of their eruptive period (first quarter), irrespective of VEI. However, ~1/3 of climactic eruptions occur in the latter half of an eruptive cycle

Where both eruptive styles exist with a single eruption phase, a common eruptive trend is from explosive to effusive activity (Fig. 2). A plot using the known durations of eruptive periods and occurrence of a climactic eruption (VEI ≥ 4 in this instance), within these periods (containing dome growth and smaller explosions) from the Domehaz database^22^, shows that almost 70% of climactic Plinian eruptions occur within the first quarter of an eruptive period (Fig. 2). The remaining 30% of the climactic eruptions occur in the latter half of their respective eruptive period, showing that there are many instances where large explosions occur during, or after dome growth (e.g., Pinatubo, Philippines, 1991, Mt Mazama, USA 2850 BC). The most common eruptive transition from explosive to effusive has been previously attributed to volatile gradients, where the first explosive phases tap the volatile-rich top of the magma body^23^ and the latter phases sample drier, degassed magmas at the bottom. However, volatile contents from melt inclusions generally show no discernible difference between effusive and explosive eruptions^17^. Higher dissolved volatile contents (in particular H_2_O) should promote faster ascent through exsolution-driven expansion^24^, as is sometimes the case^18,25–28^. However, similarly high-water contents can also lead to slow ascent rates and effusive eruptions^17,29–31^. This issue is compounded by the difficulty in gaining accurate dissolved volatile data for magmas^32–34^. Nevertheless, as volatile measurement techniques improve, the relative importance of the different volatiles (e.g., CO_2_ versus H_2_O), as well as tracking volatile saturation evolution in affecting eruptive style may prove to be insightful^19,35^.

**Fig. 3:**
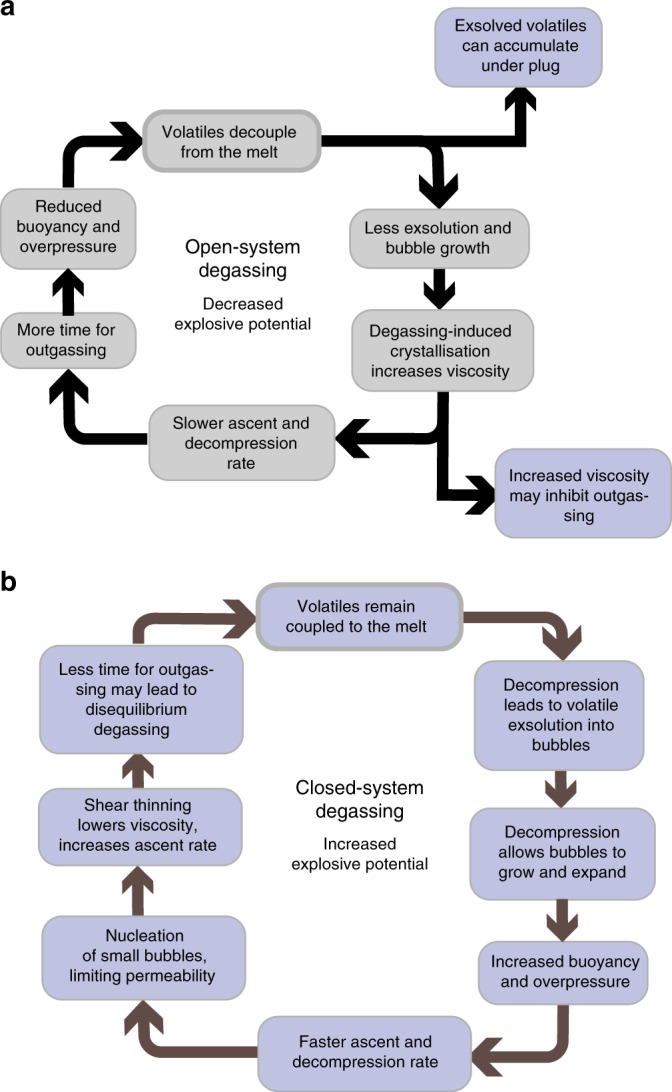
Open- and closed-system degassing feedbacks have a strong control magma ascent and eruptive style. **a** For open-system degassing, the release of volatiles will decrease the buoyancy and overpressure of the magma, leading to slower ascent and allowing more time for equilibrium degassing and outgassing. Crystallization and viscosity increase enhance this positive feedback and left unchecked, this will normally lead to slow decompression rate, thus diminishing the likelihood of overpressure building up and leading to effusive eruptions^36,73,121^. However, negative feedbacks can also occur (purple boxes in **a**), to counteract the loss of volatiles which may lead to more explosive eruptions. **b** The added buoyancy from volatile exsolution and bubble expansion will drive faster ascent, making outgassing less efficient^179–181^, which may lead to disequilibrium degassing^48^. This positive feedback is further enhanced by nucleation of small bubbles, which may limit bubble coalescence^83^. In addition, for more silicic magmas or when decompression rates are high enough, decompression-induced crystallization will be suppressed^182^. This may have two effects, first it keeps the viscosity low to enable faster ascent and second, it provides less bubble nucleation sites and thus less potential for outgassing. For some non-Newtonian magmas excess deformation of leads to shear thinning behaviour^183,184^ decreasing viscosity^185^ and thus further increasing ascent rate^186^. These feedbacks promote more explosive behaviour

As dissolved volatile contents do not appear to be the dominant control, the explosive to effusive transition within single eruptive episodes has been attributed to differences in degassing regimes (closed- versus open-system degassing; Fig. 3)^36^. The changes in degassing behaviour are related to magma ascent, which controls how efficiently gas is lost from the magma^37^. In this instance, the first explosive eruptions are driven by fast ascent speeds leading to closed-system degassing. Fast magma ascent leads to explosive fragmentation, potentially through both volatile overpressure in bubbles and high-strain rates due to rapid acceleration. Following this, lava extrusion occurs as a result of open-system degassing related to the slower ascent of the magma as the overpressure declines^38,39^. For explosive Plinian eruptions, Scandone et al.^40^ suggest that these require the development of a fully connected conduit. When this happens, magma ascent rates will depend on the decompression rate, which in turn is a function of the pressure within the magma reservoir, the location of the fragmentation surface, the viscosity, and the geometry of the conduit^41^. All four parameters will change with time. For this reason, large explosive eruptions exhibit steady Plinian behaviour for only limited periods of time (typically hours, where ‘Plinian’ is a term used for towering volcanic plumes erupted from a single vent). Plinian eruptions commonly transition to ignimbrite-forming eruptions as the shallow vent widens; alternatively, if the pressure differential driving the eruption decreases rapidly, then the eruption may become effusive or cease altogether^40^. Fluctuations between effusive and explosive behaviour during the course of eruptions can be entirely modulated by stress changes caused by the eruption, for instance, decompression during the eruption has been known to tap deeper magma bodies (e.g., Eyjafjallajokull, Iceland^42^). Further insights into this transition can also be gained from direct observations. For instance, during the 2011 rhyolitic eruptions of Cordon Caulle, Chile, the initial Plinian episode was followed by ‘effusive’ lava flows, which were in fact accompanied by mild explosive activity^43,44^. This explosive to effusive transition however does not always occur, as evidenced by the cluster of climactic explosive eruptions at the end of an eruptive period (Fig. 2), showing that there are other ways to cause transitions into explosive behaviour. This may make future eruptive activity difficult to predict in our current state of knowledge, as demonstrated by the ongoing (2017–2018) activity at Agung volcano, Indonesia.

### Magma ascent and decompression

**Fig. 4:**
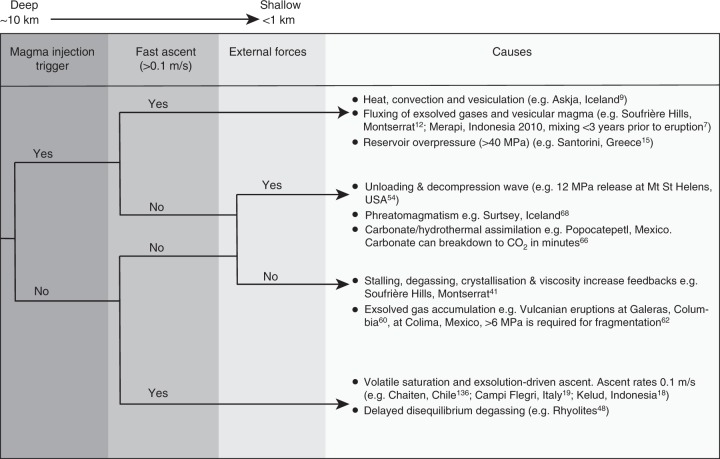
Event tree diagram showing the different processes/conditions that may lead to explosive eruptions. Slow ascent scenarios often involve transitions between explosive and effusive activity. Speed of ascent may fluctuate during an eruptive phase, this diagram refers to the average ascent rate from storage to the surface

Magma ascent and decompression rates (mostly coupled) are perhaps the most critical parameters controlling volcanic style^37^. In Fig. 4, we simplify magma ascent into fast and slow, referring to an average velocity from the storage system to the surface. In reality, magma ascent rate will vary considerably from the reservoir to the surface depending on the relative changes in vesicularity (buoyancy), exsolved and dissolved volatile content, overpressure at depth relative to surface, magma rheology and conduit geometry. We mostly refer to magma ascent rather than decompression here as magma ascent rate is more meaningful for volcano monitoring purposes.

**Fig. 5:**
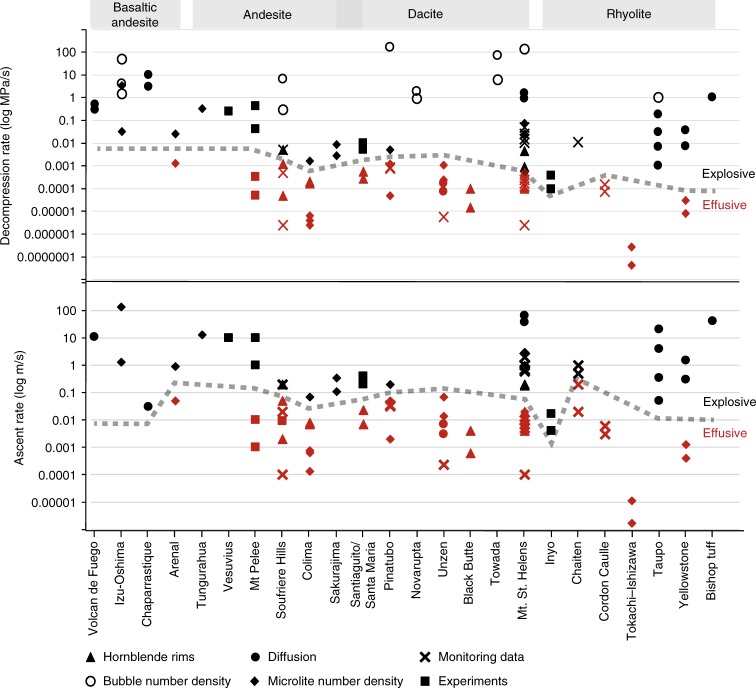
Comparison of all syn-eruptive ascent rates estimates, from multiple volcanoes ranging from basaltic andesite to rhyolites (Supplementary data 1). This plot collects ascent and decompression rate data from a range of different techniques, including microlite crystallinity, bubble number density, experiments, hornblende rims, seismicity, extrusion rate and diffusion rates. Where decompression rates were not given, these were calculated from the ascent rates using an assumed lithostatic pressure gradient of 0.025 MPa/m. Magma ascent and decompression rates are from the following sources, from left to right: Volcan de Fuego, Guatemala^187^; Izu-Oshima, Japan^188,189^; Chaparrastique volcano, El Salvador^190^; Arenal, Costa Rica^191^; Tungurahua, Ecuador^192^; Vesuvius, Italy^193^; Mt Pelee, Martinique^194^; Soufriere Hills, Montserrat^195–198^; Colima, Mexico^65,199^; Sakurajima, Japan^200^; Santiaguito/Santa Maria, Guatemala^201,202^; Pinatubo, Philippines^189,203,204^; Novarupta, USA^90^; Unzen, Japan^205–207^; Black Butte, USA^208^; Towada, Japan^189^; Mt St Helens, USA^189,209–213^; Inyo domes, USA^52^; Chaiten, Chile^136,214^; Cordon Caulle, Chile^215^; Tokachi–Ishizawa, Japan^216^; Taupo, New Zealand^217–219^; Yellowstone, USA^220,221^; and Bishop tuff, USA^222^

Despite a range of different techniques used to estimate these ascent or decompression rates (Fig. 5 and Supplementary data 1), there is a distinction between rates of effusive versus explosive eruptions. This divide seems to occur around 0.001 MPa/s, 0.1 m/s (Fig. 5); however, this is not a strong divisional boundary, but a gradational range of rates (e.g., 0.0001–0.005 MPa/s, and 0.005–0.25 m/s), where transitional and pulsatory effusive and explosive activity is more likely. For example, there are numerous places where ascent rates for explosive eruptions are low (e.g., associated with small Vulcanian eruptions at Chaparrastique, El Salvador and Colima, Mexico) and effusive ascent rates are high (e.g., rapid dome growth at Chaiten, Chile, following initial Plinian eruption). There is likely a weak correlation with composition, where lower viscosity basaltic andesites reach faster ascent rates compared to rhyolites, but this requires further investigation. We must also point out several caveats to this data set, (1) these are syn-eruptive rates and do not necessarily show magma ascent prior to eruption, (2) these are average rates, whereas magma ascent and decompression are dynamic processes, which evolve from storage to surface, (3) the plot comprises many different types of eruptions of differing scales, e.g., small Vulcanian eruptions plotted along with large voluminous Plinian eruptions of Taupo. Nevertheless, it shows that syn-eruptive magma ascent and decompression rates have a strong control on eruptive style, due to their role in governing open- versus closed-system degassing feedback cycles, which influence the extent of outgassing prior to, and during an eruption (Fig. 3).

Although the controls on magma ascent and decompression rate can be broadly identified, their relative importance is not yet clear. Sparks and Melnik^41^ suggested that magmatic ascent at Soufrière Hills volcano was linked directly to magma chamber overpressure. The magma chamber and conduit acted as energy capacitors, storing energy from elastic deformation of the wall rock, until the pressure overcame a threshold, which then drove fast ascent and explosive eruptions. Thomas and Neuberg^45^ used a suite of conduit flow models to determine the dominant factors controlling magma ascent, based on the Soufrière Hills volcano. In this study, conduit diameter and excess pressure in the magma chamber were among the dominant controlling variables, but the single most important parameter was the volatile content (dissolved + exsolved and assumed as only water). This is because volatiles lower the melt viscosity and also lead to greater exsolution and production of exsolved volatiles, thereby increasing magma buoyancy. Meanwhile, other parameters such as density and the external stress field, simulated by varying the pressure at the surface, were deemed to be less important, the latter becoming more influential at shallower levels. This is consistent with the observations that eruptions from deep magma storage, where the pressure difference between the source and the surface is highest, do not always produce explosive eruptions, and that many eruptions sourced from shallow magmas can be very explosive. Furthermore, in the cases where pressure at the surface is confined, e.g., for subglacial eruptions, explosive eruptions can also occur^46^.

Explosive eruptions are modulated in part by the conditions of bubble formation (vesiculation), which require both nucleation and growth. The exsolution of volatiles within a magma is controlled by the decompression rate, the degree of volatile saturation, availability of nucleation sites, surface tension and viscosity of the magma^47^. Therefore, when the decompression rate is high, the volatiles may not be able to degas from the magma in equilibrium with their relative solubilities. This is termed disequilibrium degassing and it may lead to volatile supersaturation and high overpressures (>100 MPa), so that when vesiculation does occur, it occurs at higher rates than equilibrium degassing and thus may increase ascent speeds and volcano explosivity^48^. Disequilibrium degassing is more common in silicic magmas, due to their higher viscosities and lower diffusivities that tend to resist bubble nucleation. Nucleation directly from silicic melt (termed 'homogeneous' nucleation) requires supersaturation pressures that are high enough to overcome the high melt-vapour surface tension (>120–350 MPa^48,49^), depending on the melt viscosity. Such supersaturation pressures are unrealistically high (often higher than inferred storage pressures (see recent review by Shea^50^) and the evidence for homogenous bubble nucleation in natural magmas is limited. In many cases, a component of 'heterogeneous' nucleation (nucleation of crystal surfaces) may be required. Clearly further studies are necessary here, such as careful petrological work along with numerical models to investigate the role of disequilibrium degassing and crystallization on ascent dynamics^51^.

### Slower magma ascent leading to explosive eruptions

Fast ascent (e.g., >0.1 m/s), where melt and exsolved gas remain coupled, will almost always lead to explosive eruptions (Fig. 5). However, the contrary is not true, as slowly ascending magmas (e.g., <0.01 m/s), can also cause explosive eruptions (e.g., Inyo volcanic chain, USA^52^). An extreme example of the explosive potential of slowly ascending magmas is provided by the 1980 eruption of Mt. St. Helens, USA. Here, 2 months before the explosive eruption, magma ascended at a rate of ~0.01 m/s into the shallow system, creating a cryptodome^40,53^. The eruption was eventually triggered by failure of the edifice because of pressure from the growing cryptodome^54^. This rapid unloading event created a downward-propagating decompression wave that caused a runaway effect of gas expansion, rapid ascent, and fragmentation of deeper-seated magma whereby a fully connected conduit was established between the surface and the deep-seated magma reservoir^40^. The Mt St Helens example demonstrates both of the two main types of fragmentation mechanisms, rapid decompression. The sustained Plinian phase of the eruption results from the other type of fragmentation that arises from the rapid acceleration of gas-rich magma. In this instance, vesiculation and bubble growth create high strain rates to cause brittle fracturing of the magma as it goes through the glass transition^55^ (Fig. 1a), the higher porosity from rapid vesiculation also helps to lower the fragmentation threshold, i.e., the pressure drop required to fragment the material^56^.

Explosive eruptions from slowly ascending magmas may also be pulsatory and modulated by competition between slow magma ascent and build-up of overpressure beneath a viscous lava plug at the top of the magma columns leading to transitions in explosive activity. Sparks and Melnik^41^ show that feedbacks between degassing and crystallization during slow ascent can increase viscosity and thus cause pressurization of the magma in the upper parts of the conduit during dome growth; this model was calibrated using patterns in tilt and seismic signals at the Soufriere Hills volcano, Montserrat. Slow ascent, coupled with sealing of pores by lava, pyroclasts, or cristobalite^57–59^ also allow exsolved volcanic gases to accumulate at shallow levels. Under these conditions, gas pressurization can be contained by the elastic deformation of the wall rocks until this overpressure exceeds the strength of the confining rock^60–62^. Failure of the capping plug causes high-intensity explosions known as Vulcanian eruptions; downward-propagating decompression waves following plug failure can rapidly evacuate the volcanic conduit to depths of a few km^63,64^. Subsequent slow re-filling of this conduit re-starts the Vulcanian eruption cycle. Confirmation of this heuristic model is provided by measurements of emitted volatiles, particularly SO_2_, where gas exhalations can be correlated with the repose times between eruptions^65^.

### External forces

Transitions in explosivity may be influenced by external factors independent of other magmatic variables (Fig. 4 and Table 1). These usually occur at the shallow level (<1 km) or at the surface. Rapid changes to the edifice such as sector collapses can trigger top-down fragmentation as discussed in the Mt St. Helens eruption above. Another example is the assimilation of external materials such as carbonate, which can also promote explosive volcanism through the formation and rapid expansion of CO_2_ bubbles, these exsolved volatiles, when coupled to the melt may drive faster ascent and thus increase explosivity at volcanoes such as Merapi, Popocatepetl and Vesuvius (Table 1^66,67^). Sudden explosive activity can also be caused by external water and magma interaction, driven primarily by the volumetric expansion as the water is superheated, leading to explosive fragmentation^68^. At the most explosive end, more than 30% of the available thermal energy can be converted into mechanical energy, most of which is emitted as shock waves, which may enhance fragmentation within the conduit and vent^69^.

## Keeping magma from erupting explosively

The acceleration, fragmentation and explosive eruption of magma are powered by the exsolution of volatiles dissolved in the melt and the expansion of these gases once they form bubbles^70^. Exsolution accompanies ascent and decompression because the solubility of volatiles decreases with decreasing pressure, though increases in temperature caused by deformation^71^ or recharge may also lead to exsolution^8^. Key to preventing explosive eruption is thus to keep pressure within bubbles from getting so high that the melt around bubbles ruptures^72^ or to remove gases from ascending magma^73^.

The escape of gas from rising silicic (high viscosity) magmas requires that the magma is permeable. Over the past decades, great progress has been made in measuring and modelling permeability, and more recently recognizing that permeability is a highly transient property^74–79^. Prior to fragmentation or brittle failure, magmas become permeable as bubbles become connected. Permeability is an evolving quantity, increasing as bubbles grow and coalesce and the magma deforms, and decreasing as gas loss causes bubbles to collapse (Fig. 3b^36,80–83^). Permeability development is moderately sensitive to decompression rate, but strongly affected by variations in melt composition (viscosity) and crystallinity^84,85^. The signatures of bubble growth and gas loss are recorded in the textures of volcanic rocks^86^. Extensive measurements of permeability on quenched magmas^87^ suggest that magma permeability exerts a leading order control on whether magma is able degas fast enough during ascent to avoid fragmenting^88^.

**Fig. 6:**
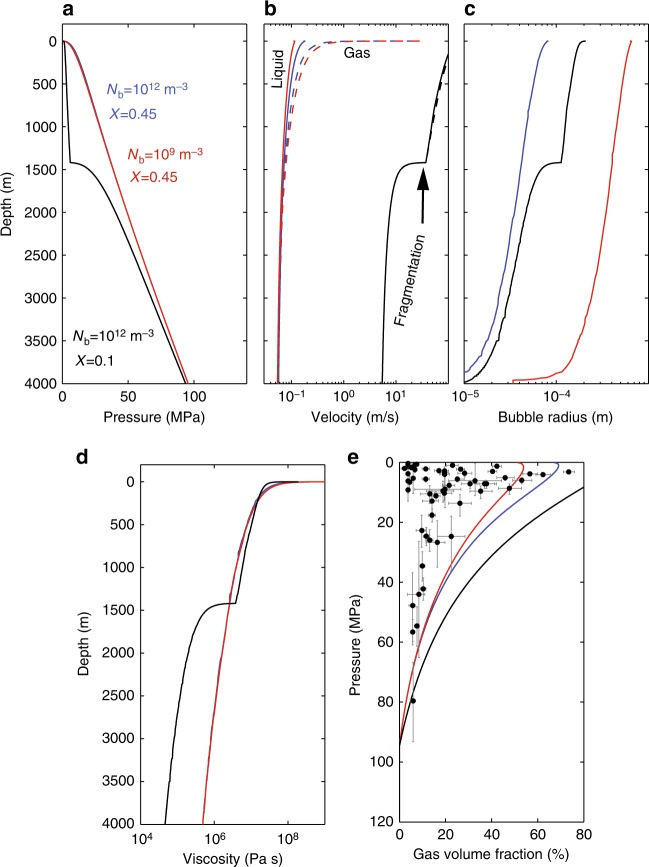
Comparing the properties during ascent of hydrous leucogranite with different bubble number densities (N_b_) and crystal contents (X). The model simulates magma ascent and gas escape, computed using the steady one-dimensional model of Degruyter et al^83^., showing how **a** pressure, **b** melt (solid curves) and gas (dashed curves) velocities, **c** bubble radius and **d** magma viscosity evolve during ascent; **e** shows how vesicularity varies with pressure. Also, included vesicularity and the inferred pre-eruptive depth of clasts evacuated from the conduit during the 1997 Vulcanian eruptions at Soufrière Hills Volcano, Montserrat^223,224^. These samples are thought to capture conditions during ascent for the dome-forming eruptions^224^. Errors bars from Burgisser et al.^224^ account for uncertainty in measured water content (pressure) and vesicularity. Note that the volume fraction of exsolved gas in the clasts is lower than expected when gas is allowed to escape from the rising magma. The effect of crystal fraction on viscosity is based on the model of Costa^178^ and the melt viscosity model is that of Hess and Dingwell^177^. Fragmentation occurs at a gas volume fraction of 85%. In the model, crystals do not grow, and N_b_ does not change during ascent. Homogeneous bubble nucleation and equilibrium degassing are assumed. Volatile content at depth is 4 weight %. Temperature is 886 °C. The percolation threshold for gas flow through the magma is zero, tortuosity factor is 3, bubble throat to radius ratio is 1, and the friction coefficient for gas flow through the magma is 10, values as used in Degruyter et al.^83^

A combination of slow ascent or decompression, and efficient gas loss (high permeability) promote effusive activity^89,90^. Figure 6 shows the consequences of degassing by porous gas flow on magma ascent and properties within the conduit. Ascent is computed using the equations presented in Degruyter et al.^83^ (see methods). The model solves for the one-dimensional two-phase vertical flow of magma and gas, assumes equilibrium exsolution, includes a model for the dependence of permeability on bubble size and gas volume fraction, and accounts for the pressure dependence of water solubility and the effect of dissolved water and crystals on melt viscosity. We assume there is no critical porosity (percolation threshold) to initiate gas flow. Further details and model parameters are summarized in the figure caption. Effusive eruption is promoted by a lower number density of bubbles that leads to larger bubbles (Fig. 6c) and hence higher permeability and greater gas velocity (Fig. 6b) and loss (Fig. 6e). Higher viscosity, in the examples shown in Fig. 6 provided by higher crystallinity, leads to slower magma ascent and hence permits more time for gas to escape from the rising magma. Pressure decreases during ascent, and the rate of pressure decrease is controlled by the resistance to ascent, which involves feedbacks between exsolution, viscosity and gas escape ('outgassing'). Once magma fragments, it leads to lower pressures at a given depth within the conduit (in the fragmented magma) and thus exsolution and higher melt viscosities.

Permeability is an even more dynamic quantity after magma undergoes brittle failure. High strain rates can also fracture magma^54,55^ and this fracturing can promote further nucleation^91^. Fractures can be transient in viscous magma because viscous deformation allows cracks to anneal and heal^92–94^ but strain is not immediately localized to fractures, permitting efficient outgassing^95^. Transient fractures, can also create temporary pathways that can transport ash, in addition to gas. A signature of these transient processes is preserved in fragment-filled veins called 'tuffisites^43,58,96–98^' or texturally banded rocks in lava domes^99–101^. Weak venting through such pathways can also accompany otherwise effusive eruptions^44,102^. Magma fracture may occur preferentially at the margins of conduits where strain rates are highest^103,104^ and can be magnified by shear heating and strain localization^77,105–110^. Magma fracture and welding is commonly preserved at the microscale in the form of fractured crystals, cuspate vesicles and xenocrystic (foreign) material^111–113^. Accounting for feedbacks between fracturing and degassing in models such as that shown in Fig. 6 is complicated by our limited understanding of permeability and welding, processes that are time dependent and sensitive to the poorly constrained fragment size distributions^114,115^.

Gas escape is further enhanced if the surrounding country rock is permeable, a property ignored in Fig. 6, in which case the degassing efficiency is controlled by both the permeability anisotropy, which controls the directionality of gas escape, the wall rock permeability, and the ambient pressure in the wall rock, e.g., lithostatic, hydrostatic or atmospheric^73,116–118^. The deformation that accompanies magma ascent can promote failure in the surrounding rocks and hence gas escape^119^, delivering gases to the hydrothermal systems surrounding magma bodies^120^. The transport of gases, and their interaction with the surrounding rock or domes that cap conduits, create minerals that act to seal cracks so that country rock and dome permeability will also be transient^116,121^.

Although conduit geometry, permeability and crustal properties are clearly important factors in controlling magma ascent and outgassing, due to the difficultly in constraining these, only relatively few studies exist and these mostly focus on modelling^122,123^. Conduit wall permeability can alter significantly when progressively heated by reducing open porosity and thus limiting outgassing during ascent^117^. Conduit geometry has a fundamental effect on magma ascent, and the conduit may widen to accommodate higher magmatic overpressures, discharge rates and higher viscosity magma, and thus may contribute to the positive feedback mechanism to increase magma ascent rate^124,125^. However, more integrated field studies on exposed volcanic conduits, along with petrology and numerical simulations would be beneficial in this area^96,126,127^.

The interacting processes described above involve both positive and negative feedbacks between magma ascent and gas loss. Positive feedbacks occur when faster ascent enhances bubble nucleation, which in turn produces smaller bubbles and reduced permeability^83^, and shear deformation that causes heating and vesiculation^71^. Negative feedbacks include the sealing of melt, dome and country rock fractures as a result of gas loss^59,128^; heating of the wall rock to create a viscous 'brake' by inhibiting frictional slides^109,129^; deformation during ascent that increases permeability and gas loss by promoting bubble coalescence; and crystallization driven by gas loss that increases magma viscosity and slows ascent. The combination of positive and negative feedbacks is one way to generate episodicity or even periodicity in eruption rate^130^.

## Visualizing the controls on eruptive styles

**Fig. 7:**
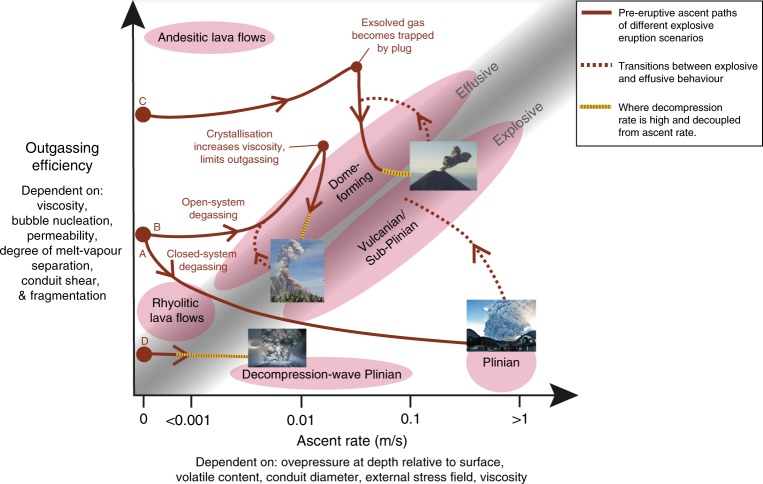
Schematic plot, where an diffuse threshold distinguishes effusive from explosive eruptions. Points **a**–**d** represent different eruptive scenarios: **a** = Ascent controlled (bottom-up), **b** = Viscosity controlled, **c** = Exsolved gas accumulation and plug controlled, and **d** = Decompression-wave controlled. The red lines are pre-eruptive ascent paths and do not represent transitions between effusive and explosive behaviour, where the eruption photo represents a magma reaching the surface. The starting position on the outgassing efficiency axis depends on the magma rheology (composition and crystallinity) and permeability. Note that this figure is schematic, the ascent rates are based on Fig. 3 (syn-eruptive averages). However, currently no data exists on how ascent and decompression rates evolve during transit from storage to surface. In addition, due to the complex and variable nature of magmatic outgassing, and how this changes with crystallization, viscosity and bubble nucleation during ascent, data on this parameter are currently lacking. Nevertheless, quantifying these parameters during the magma’s transit from storage to surface in the future, may prove crucial for understanding the controls on eruptive style and where in the crust these controls are most important. Eruption photos in this figure are sourced from Chaiten in 2008 (Daniel Basualto), Soufrière Hills, 1997, Volcan de Colima (Mike Cassidy) and Mt St. Helens in 1980

Despite the range of different properties (Table 1), processes (Figs. 1 and 3), and scenarios (Fig. 4), controls on silicic eruption styles can, we propose, be characterized more simply, by combining some variables schematically: Fig. 7 shows changes in ascent rate from storage to surface as a function of outgassing efficiency. The ascent rate is largely a product of the overpressure, crustal stress field, conduit radius, viscosity and buoyancy (driven by volatile exsolution and decompression) of the magma, while outgassing efficiency is dominantly controlled by the viscosity, pressure or solubility, time (linked inherently to ascent rate), bubble nucleation/coalescence, permeability of magma and conduit wall rock, and fragmentation processes . The graded distinction between effusive and explosive regimes in Fig. 7 combines most of the properties laid out in Table 1 and highlights that some magmas will inherently be able to outgas efficiently. For example, a low viscosity or permeable magma will require faster ascent or decompression rates to generate an explosive eruption, as faster decompression inhibits outgassing prior to, and during an eruption. In contrast, for a magma that has a low outgassing efficiency, such as a high-viscosity rhyolite, an explosive eruption may occur at slower ascent speeds relative to lower viscosity magmas, since it is likely that the volatiles will be retained within the magma during closed-system degassing (Fig. 7). This is reflected in the different starting positions on the outgassing efficiency axis for the various eruptive scenarios (A-D). Andesite melts are inherently more efficient at outgassing due to their lower viscosity relative to rhyolites, and they also commonly contain more crystals, which may help to channelize exsolved volatiles to further enhance outgassing efficiency via veining and capillary fracturing^85,131,132^. However, magmas rich in crystals have also been proposed to reduce outgassing^133,134^, and there is likely a ‘sweet-spot’ of enhanced buoyancy-driven volatile outgassing (40–50% volatiles outgas) when crystallinities are between 40–70%^135^.

Also plotted are the different explosive eruption scenarios (as referred to in Fig. 4), each with their dominant control. All ascent paths for the different scenarios begin from a stationary magma body in storage and continue to the point of eruption at the surface (eruption photo on Fig. 7). For eruptive scenarios that endure closed-system degassing during magma decompression and volatile exsolution, the exsolved volatiles remain coupled to the melt (Scenario A). Uninterrupted, this closed-system degassing feedback (Fig. 3b), driven from the ‘bottom-up’ may lead to a runaway process that ultimately leads to explosive fragmentation in a Plinian eruption (e.g., Chaiten, 2008; Scenario A or ‘Ascent controlled’^136^. Where open-system degassing occurs, such as at the start of Scenarios B and C (Fig. 7), the opposite feedback will occur (Fig. 3a), making an effusive silicic eruption more likely (section 3). However, this cycle can be broken in two ways: either the viscosity increases, via decompression crystallization and volatile exsolution, thereby dramatically reducing the outgassing efficiency (e.g., Vulcanian and sub-Plinian eruptions of Soufrière Hills volcano; Scenario B or ‘Viscosity controlled’^137^); or the exsolved volatiles become sealed, accumulate, and then experience high decompression rates once the plug is broken (e.g., Scenario C or ‘Exsolved gas accumulation and plug control,’ e.g., Galeras^60^). These scenarios are common in dome-forming volcanoes that have frequent Vulcanian or sub-Plinian eruptions and where activity can rapidly fluctuate and transition between effusive–explosive eruptions (dashed lines in Fig. 7), or even occur contemporaneously^44^. Scenario D highlights the ‘top-down’ process (Scenario D or ‘Decompression wave-controlled’ in Fig. 7) which can be largely independent of ascent rate and solely controlled by decompression, via a downward-propagating decompression wave, triggering explosive fragmentation (yellow dotted lines in Fig. 7) (e.g., Mt. St. Helens^54^).

These eruptive scenarios and type examples show that there is a fine balance between open- and closed-system degassing at the start of magma ascent from storage. Once one mode of degassing is preferred at this early stage this may, in some instances, trigger the aforementioned feedbacks and thus dictate the style of the initial eruption phase. Closed-system degassing could occur either when early outgassing is inhibited by high viscosity or low degrees of melt/vapour segregation^138^ thus increasing magma ascent rate, or alternatively when fast initial magma ascent (driven by overpressure, buoyancy, decompression, etc^41^) limits outgassing. This ‘chicken or the egg’ situation is often governed by processes and conditions at depth and thus it is particularly important to constrain the magmatic storage conditions. This includes the properties, and processes at the deep storage level, such as the rheology, overpressure, initial outgassing mechanisms, exsolved plus dissolved volatile contents and the role of wall rocks, as these may be critical for driving the initial feedbacks and thus governing eruptive behaviour.

## Remaining knowledge gaps

This review highlights recent progress in understanding the dominant factors that control volcano explosivity. However, because of interactions between complex processes and multiple interrelated parameters, open questions remain. In this section, processes and parameters that are poorly understood will be discussed, along with where potential future research should be directed. We then highlight how the most important parameters can be monitored to enable eruptive style to be forecast.

Of the different explosive eruption scenarios displayed in Fig. 7, some are better understood than others. For instance, decompression waves (top-down eruptions) have been replicated in the lab^139^, while moderate sized eruptions (VEI 2-3) occur relatively frequently and thus have benefited from direct observations and measurement (e.g., Soufrière Hills^140^). However, the least well understood are the most explosive, the Plinian eruptions (driven from the bottom-up). The fact that the climactic Plinian eruptions do not only occur at the start of an eruptive period, but also at the end (Fig. 2), highlights that these eruptions are not simply driven by larger gas accumulation and plug-controlled eruptions, but are more complex, likely driven by a combination of different processes^141^. This means that numerical conduit flow models used to simulate Plinian eruptions are still limited^142^. Most conduit flow models assume isothermal, equilibrium degassing, steady-state flow with constant conduit geometry, inlet pressure, and neglect mechanical and thermal feedbacks with the surrounding crust^142^. These assumptions, despite being partly unrealistic, are used because the processes that govern conduit evolution are complex and cannot always be formulated into a set of equations with known parameters. This is an area where a new generation of models coupled to magma reservoir processes are required, together with better constraints on physical processes and parameters involved in the models.

The presence of exsolved volatiles (gas bubbles) in the magma is known to increase buoyancy and ascent speed^143^, factors that may lead to higher explosivity^144^. However, Degruyter et al.^145^ suggest that during recharge events the presence of exsolved volatiles in the magma reservoir may lower explosivity. They attribute this to an increase in magma reservoir compressibility, dampening pressurization, which allows for a significant amount of recharge and heating before eruption, thus enhancing conduit outgassing and slowing down ascent. Evidently the role of exsolved volatiles requires further testing and constraining to understand its role in ascent dynamics, using volcanic gas measurements^30^, analogue and petrological experiments to develop petrological indicators^133,143^, and numerical modelling^145^.

## Forecasting eruptive style

Understanding the dominant parameters and processes that affect eruptive style and striving to measure these by volcano monitoring, may be the most promising way to improve forecasts of eruption style and explosivity. However, we face a number of challenges highlighted in the following paragaraphs.

Eruptive transitions that occur within a single phase (e.g., scenarios B, C and D; Fig. 7), are inherently difficult to forecast via monitoring. In these instances, changes can be fast (minutes to hours) and they can be modified by shallow-conduit and vent dominated processes such as conduit shear, development of anisotropy, permeable outgassing, dome collapses and the formation of tuffisites. Here, new work on degassing and time-dependent evolution of permeability and strength during deformation may help in forecasting time intervals where overpressure may build to generate explosions^146^. Shallow processes such as these may be related to short deformation cycles, and long-period and hybrid seismicity thought to be related to excess fluid pressure^137^.

Eruption transitions that occur across different eruptions at the same volcano (e.g., scenario A, Fig. 7) are dominantly governed from the bottom-up and can be linked to processes occuring within the magmatic plumbing system, and thus offer more promise in forecasting volcanic behaviour. Seismicity, deformation and gas measurements may be used to interpret conditions in the magma plumbing system. For instance, long period earthquakes, seismic velocity changes and ‘drumbeat’ seismicity have been used to detect magmatic ascent^109,119,147^; pre-eruptive, InSAR and tilt data were linked to the rate of pressure change and resulting explosivity of an eruption^148,149^; and increases in CO_2_ relative to SO_2_ phases have been recorded before some explosive eruptions^150,151^. However, those monitoring techniques require much refinement before volcanologists can, in near real-time predict future behaviours of a given volcano. Combining these measurements with constraints from petrology, fieldwork and modelling will be key to diagnosing the future dominant eruptive styles for each volcano.

Under most circumstances, rapid magma ascent requires a pre-existing pathway, or conduit. Conduit formation and evolution are challenging to study. The duration of shallow seismicity weeks to months prior to many volcanic eruptions suggest that this is in part related to conduit creation, although various other processes can create seismicity (e.g., White and McCausland^152^ and references therein). The data compilation by White and McCausland^152^ reveals that the onset of eruptive activity is commonly phreatic and is closely followed by a magmatic eruption. The type of pre-eruptive seismicity is also key as earthquake type may change from high frequency (rock breakage) to low frequency (involvement of fluids) as the eruption initiation is approached^153^. These patterns form the basis of the Failure Forecast Method for predicting eruptive activity, which is predicated on patterns of exponentially increasing rates of seismicity as a failure threshold is approached^154–156^. Despite the conceptual appeal of the model, successful applications of the technique have been limited, in part because of the material complexity of volcanic edifices^157^. A modified approach has been suggested by Bell et al.^158^, which includes appropriate error distributions to the forecast method. In addition to seismicity, magma overpressure can be monitored geodetically^159^ and degassing efficiency can be assessed using pre- and post-eruptive volatile emissions^121^. Together these observations suggest that under many conditions, each new eruption requires construction of a new conduit, and may involve interaction with shallow hydrothermal systems, and that the actual trigger for explosive eruption is often difficult to identify.

Multiple existing monitoring datasets have shown that explosive eruptions are often preceded by short but intense periods of unrest, with increased rates and magnitudes of both seismicity and deformation^160^. Hence, future efforts will benefit from real-time multi-parameter modelling and hazard alert systems. Once an eruption has started, constraining lava effusion rate and volume of mass erupted using thermal imagery, radar and aerial photogrammetry has proven to be useful in monitoring and forecasting changes in explosivity^104,161,162^. Future studies such as these, along with integrated studies linking monitoring data with geological, petrological, and numerical modelling^163–165^ will improve explosivity forecasting. In addition, drilling upper magmatic reservoirs and adjacent wall rocks will provide in situ information that can be linked directly to geophysical measurements^166–170^.

Finally, collating previous eruptive records^22,171^ and the development and analysis of large volcanic monitoring datasets, with the co-operation of multiple observatories around the world, e.g., WovoDat^172^ and Global Volcanism Program^3^, will be critical if we are to more accurately forecast whether an impending eruption will be effusive or explosive.

## Methods

### Equations describing magma ascent in the conduit

We treat magma as two distinct phases: gas and melt + crystals (the latter hereafter called magma). These two phases can move with respect to each other and are coupled through drag forces and equations of state. The formulation of the governing equations and closure models are summarized in Degruyter et al.^83^ that in turn builds on models developed by Kozono and Koyaguchi^173,174^ (Fig. 6).

Conservation of mass for the melt and gas are, respectively,

1 $$\frac{{{\rm{d}}\left( {\rho _mu_m\left( {1 - \phi } \right)} \right)}}{{{\rm{d}}z}} = - \frac{{{\rm{d}}n}}{{{\rm{d}}z}}q$$

and

2 $$\frac{{{\rm{d}}\left( {\rho _gu_g\phi } \right)}}{{{\rm{d}}z}} = \frac{{{\rm{d}}n}}{{{\rm{d}}z}}q.$$

Subscripts *m* and *g* denote the magma and gas phases respectively, *ρ* is density, *ϕ* is the gas volume fraction a*n*d *n* the gas mass fraction, *q* is the total mass flux, *u* is velocity and *z* is the vertical coordinate.

Conservation of momentum for the gas and melt are, respectively,

3 $$\rho _mu_m\left( {1 - \phi } \right)\frac{{{\rm{d}}u_m}}{{{\rm{d}}z}} = - \left( {1 - \phi } \right)\frac{{{\rm{d}}P}}{{{\rm{d}}z}} - \rho _m\left( {1 - \phi } \right)g + F_{mg} - F_{mw}$$

and

4 $$\rho _gu_g\phi \frac{{{\rm{d}}u_g}}{{{\rm{d}}z}} = - \phi \frac{{{\rm{d}}P}}{{{\rm{d}}z}} - \rho _g\phi g - F_{mg} - F_{gw}$$

where *P* is pressure (assumed the same in both phases), *g* is gravity, *F*_*mg*_ is the friction between gas and magma, *F*_*mw*_ and *F*_*gw*_ are the friction between the magma and gas and the conduit walls, respectively.

We neglect energy conservation, though temperature *T* will enter through its effects of material properties such as gas density and melt viscosity.

The magma phase is assumed to be incompressible and the gas density is computed from the ideal gas law

5 $$\rho _g = \frac{P}{{RT}}$$

where R is the specific gas constant. Solubility is approximated by

6 $$n = \frac{{c_0 - sP^{1/2}}}{{1 - sP^{1/2}}}{\mathrm{for}}\;n \ge 0$$

where *c*_0_ is the initial (dissolved) water content of the magma, and *s* is the saturation constant.

Closure of the conservation of mass and momentum equations requires models for the friction terms. Prior to fragmentation, we assume resistance to ascent is governed by Stokes flow and hence

7 $$F_{mw} = \frac{{8\mu _mu_m}}{{r_c^2}}\;{\mathrm{for}}\;\phi \le \phi _f$$

and *F*_*mw*_ = 0 for *ϕ* > *ϕ*_*f*_ where *ϕ*_*f*_ is the gas volume fraction at fragmentation, *r*_*c*_is the conduit radius, and *μ*_*m*_ is the magma viscosity. In contrast, *F*_*gw*_ = 0 for *ϕ* ≤ *ϕ*_*f*_ and

8 $$F_{gw} = \frac{\lambda }{{4r_c}}\rho _gu_g^2\;{\mathrm{for}}\;\phi > \phi _f$$

where *λ* is a drag coefficient controlled by the roughness of the conduit.

The gas-magma coupling described by *F*_*mg*_ is more complex as it depends on the geometry of the pore space and pressure gradients prior to fragmentation, and how particles are coupled to the gas after fragmentation. Here we use the model of Yoshida and Koyaguchi^175^ to smooth the transition between non-fragmented and fragmented magma over an interval $\phi _f < \phi \le \phi _t$ and $t = (\phi - \phi _t)/(\phi _f - \phi _t)$:

9 $$F_{mg} = \left\{ {\begin{array}{*{20}{c}} {\left[ {\frac{{\mu _g}}{{k_1}} + \frac{{\rho _g}}{{k_2}}\left| {u_g - u_m} \right|} \right]\phi \left( {1 - \phi } \right)\left( {u_g - u_m} \right){\mathrm{for}}\phi \le \phi _f} \\ {\left[ {\frac{{\mu _g}}{{k_1}} + \frac{{\rho _g}}{{k_2}}\left| {u_g - u_m} \right|} \right]^{1 - t}\left[ {\frac{{3C_D}}{{8r_a}}\rho _g\left| {u_g - u_m} \right|} \right]^t\phi \left( {1 - \phi } \right)\left( {u_g - u_m} \right){\mathrm{for}}\phi _f < \phi \le \phi _t} \\ {\frac{{3C_D}}{{8r_a}}\rho _g\phi \left( {1 - \phi } \right)\left( {u_g - u_m} \right)\left| {u_g - u_m} \right|{\mathrm{for}}\phi > \phi _t} \end{array}} \right.$$

where *C*_*D*_ is a drag coefficient and *r*_*a*_ the size of fragments after fragmentation. *k*_1_ and *k*_2_ are the Darcian *k*_1_ and inertial *k*_2_ permeabilities, respectively, in Forcheimer’s law

10 $$\frac{{{\rm{d}}P}}{{{\rm{d}}z}} = \frac{{\mu _g}}{{k_1}}u_g + \frac{{\rho _g}}{{k_2}}u_g^2.$$

For the permeabilities, we use the model of Degruyter et al.^176^

11 $$k_1 = \frac{{\left( {f_{tb}r_b} \right)^2}}{8}\phi _c^m$$

and

12 $$k_2 = \frac{{f_{tb}r_b}}{{f_0}}\phi _c^{\frac{{1 + 3m}}{2}}$$

where *r*_*b*_ is the bubbles radius and *f*_*tb*_ is the ratio of the throat radius connecting adjacent bubbles to the bubble radius, and *ϕ*_*c*_ is the connected porosity that we relate to the tortuosity *τ* using Archie’s law

13 $$\tau ^2 = \phi _c^{1 - m}$$

where *m* is a fitting constant. The bubble radius is calculated from the number of bubbles per unit volume *N*_*d*_ and gas volume fraction^100^

14 $$r_b = \left( {\frac{\phi }{{\frac{{4\pi }}{3}N_d(1 - \phi )}}} \right)^{1/3}$$

For the magma viscosity we combined a model for the effects of temperature and dissolved water on melt viscosity *μ* (*C,T*)^177^ with a model for the effects of crystals *θ* (*χ*)^178^, where *χ* is the crystal volume fraction

15 $$\mu _m = \mu \left( {C,T} \right)\theta \left( \chi \right).$$

## Electronic supplementary material

Description of Additional Supplementary Files

Supplementary Data 1
