# Supplementary material for: Controls on explosive-effusive volcanic eruption styles
Source: Nat Commun. 2018 Jul 19;9:2839. doi: 10.1038/s41467-018-05293-3 (PMC6053376; doi:10.1038/s41467-018-05293-3)
Supplement: Supplementary file 1 — Description of Additional Supplementary Files [file 41467_2018_5293_MOESM1_ESM.pdf]

Description of Additional Supplementary Files

File Name: Supplementary Data 1

Description: A collation of syn-eruptive decompression and ascent rates from basaltic andesite to rhyolites compositions from the literature, full references for these can be found in the main article. Some decompression rates have been recalculated from ascent rate an assumed lithostatic gradient, which are highlighted in yellow.
